# Levetiracetam in the Treatment of Epileptic Seizures After Liver Transplantation

**DOI:** 10.1097/MD.0000000000001350

**Published:** 2015-09-25

**Authors:** Chih-Hsiang Lin, Chao-Long Chen, Tsu-Kung Lin, Nai-Ching Chen, Meng-Han Tsai, Yao-Chung Chuang

**Affiliations:** From the Department of Neurology (C-HL, T-KL, N-CC, M-HT, Y-CC); Liver Transplantation Program and Department of Surgery (C-LC); Center for Translational Research in Biomedical Sciences, Kaohsiung Chang Gung Memorial Hospital and Chang Gung University College of Medicine (C-LC, Y-CC); Department of Biological Science, National Sun Yet-sen University (Y-CC); and Department of Neurology, Faculty of Medicine, College of Medicine, Kaohsiung Medical University, Kaohsiung, Taiwan (Y-CC).

## Abstract

After liver transplantation, patients may develop seizures or epilepsy due to a variety of etiologies. The ideal antiepileptic drugs for these patients are those with fewer drug interactions and less hepatic toxicity. In this study, we present patients using levetiracetam to control seizures after liver transplantation.

We retrospectively enrolled patients who received levetiracetam for seizure control after liver transplantation. We analyzed the etiology of liver failure that required liver transplantation, etiology of the seizures, outcomes of seizure control, and the condition of the patient after follow-up at the outpatient department. Hematological and biochemical data before and after the use of levetiracetam were also collected.

Fifteen patients who received intravenous or oral levetiracetam monotherapy for seizure control after liver transplantation were enrolled into this study. All of the patients remained seizure-free during levetiracetam treatment. Two patients died during the follow-up, and the other 13 patients were alive at the end of the study period and all were seizure-free without neurological sequelae that interfered with their daily activities. No patients experienced liver failure or rejection of the donor liver due to ineffective immunosuppressant medications. The dosage of immunosuppressants did not change before and after levetiracetam treatment, and there were no changes in hematological and biochemical data before and after treatment.

Levetiracetam may be a suitable antiepileptic drug for patients who undergo liver transplantation due to fewer drug interactions and a favorable safety profile.

## INTRODUCTION

Seizures occur in 6% to 8% of patients who undergo a liver transplantation.^1,2^ The causes of the seizures have been reported to be due to neurotoxicity induced by septic and metabolic encephalopathy, immunosuppressant medications, central nervous system infections, and cerebrovascular complications.^3^ In addition, patients who undergo a liver transplantation may also have preexisting epilepsy. Thus, choosing an antiepileptic drug (AED) for these patients remains a considerable challenge, especially for those with the concomitant use of immunosuppressants.^4,5^ The use of liver enzyme-inducing AEDs such as phenytoin, phenobarbital, and carbamazepine, may increase the activity of cytochrome P450 enzymes, resulting in a decrease in the plasma concentration of immunosuppressants and therefore a decrease in their effectiveness.^5^ Furthermore, AED side effects may have a negative impact on the liver transplant tissue.^6,7^ Most AEDs are metabolized through hepatic biotransformation, and any disruption during this process will lead to accumulation or production of toxic compounds, which may increase the risk of hepatotoxicity.^8^ Furthermore, patients who undergo a liver transplantation may have other medical conditions, such as infections. The subsequent use of antibiotics may change the serum level of AEDs, which may result in recurrence of the seizures.^5,9^

Levetiracetam (LEV) is a newer-generation AED that is mostly excreted unchanged by the kidneys, and the metabolism is not hepatic, but occurs primarily in the blood by hydrolysis.^10^ It is also an effective AED in terms of controlling seizures, including refractory epilepsy and acute symptomatic seizures,^11,12^ and it has been reported to have fewer drug interactions than the currently known medications.^4,13^ Due to the advantages of fewer drug interactions and nonhepatic metabolism, the present study reports a series of cases with epileptic seizures after liver transplantation who received LEV monotherapy, with the aim of elucidating the role of LEV in the treatment of these patients.

## PATIENTS AND METHODS

### Subjects

This is a single-center retrospective study. We use the medical data system of our hospital to identify the patients who used LEV for seizure control after undergoing a liver transplantation from January 2008 to December 2013. The Chang Gung Medical Foundation Institutional Review Board approved the study protocol.

The Liver Transplantation Program and Department of Surgery in Kaohsiung Chang Gung Memorial Hospital is one of the most advanced centers in Taiwan, with the highest number of patients and survival rate. It is also one of the leading centers for living donor liver transplantation in Asia, according to the journal Transplantation. The center is also recognized as a training center for liver transplantation by the Henri Bismuth Hepatobiliary Institute in France and the International Hepato-Pancreato-Biliary Association.

We reviewed the charts of the patient and recorded data on the reason for liver transplantation, interval of seizures after the transplantation, the biochemical data before and after the use of LEV, the dose of LEV used during admission and after follow-up at our outpatient department, concurrent use of immunosuppressant medication, and the outcome after discharge. All of the patients’ seizures were diagnosed by a neurologist who recorded the characteristics of the seizures including the etiology and semiology. All patients were followed until December 2013. Before the operation for liver transplantation, all patients had signed the inform concern that they agree the clinical detail be used in future clinical study.

### Statistical Analyses

A paired-sample *t* test was conducted to evaluate the changes of continuous variable before and after the use of LEV. These variables include hematological, biochemical, and dosage of the immunosuppressant agents

All statistical analyses were performed using the Statistical Package for Social Science (SPSS, version 11.0 for Windows; Chicago, IL). A *P* value of <0.05 was considered to be statistically significant.

## RESULTS

From January 2008 to December 2013, 958 patients received liver transplantation. Among them, 38 experienced seizures and 15 used LEV for seizure control. The demographic data of these 15 patients (eight males and seven females) are shown in Table 1. The mean age at receiving a liver transplantation was 54.1 ± 15.1 years (mean ± standard deviation, range from 12 to 66 years), and the age at seizure onset was 54.9 ± 15.6 years (range from 12 to 69 years). The interval of seizures after liver transplantation was 308.9 ± 466.1 days (range from 1 to 1534 days). The underlying reasons for liver transplantation were hepatocellular carcinoma with hepatitis (n = 4), chronic hepatitis (n = 8), neonatal hepatitis (n = 1), drug-induced hepatic failure (n = 1), and primary sclerosing cholangitis (n = 1). The etiology of the seizures included intracerebral hemorrhage (n = 4), acute cerebral infarction (n = 3), septic encephalopathy (n = 7), hepatic encephalopathy (n = 3), hepatorenal syndrome (n = 1), electrolyte imbalance (n = 1), and history of epilepsy (n = 1). The seizure type included partial seizures and secondary generalization (n = 8), and generalized convulsive seizures (n = 7). All patients received intravenous or oral LEV monotherapy for seizure control. The loading dose of LEV was 466.7 ± 441.9 mg (range from 250 to 2000 mg), and the maintenance dose was 892.9 ± 487.5 mg/day (range from 500 to 2000 mg/day). All 15 patients were seizure-free during LEV treatment. Two patients died during the follow-up, one due to severe infection (Patient 6) and the other due to ampullary carcinoma (Patient 8). The other 13 patients were still alive at the end of study period and were seizure-free without neurological sequelae that interfered with their daily activities. Of these 13 patients, 5 withdrew from LEV, as they were seizure-free. The maintenance dose of LEV used by the remaining 7 patients was 839.3 ± 580.6 mg/day (range from 250 to 2000 mg/day).

**TABLE 1:**
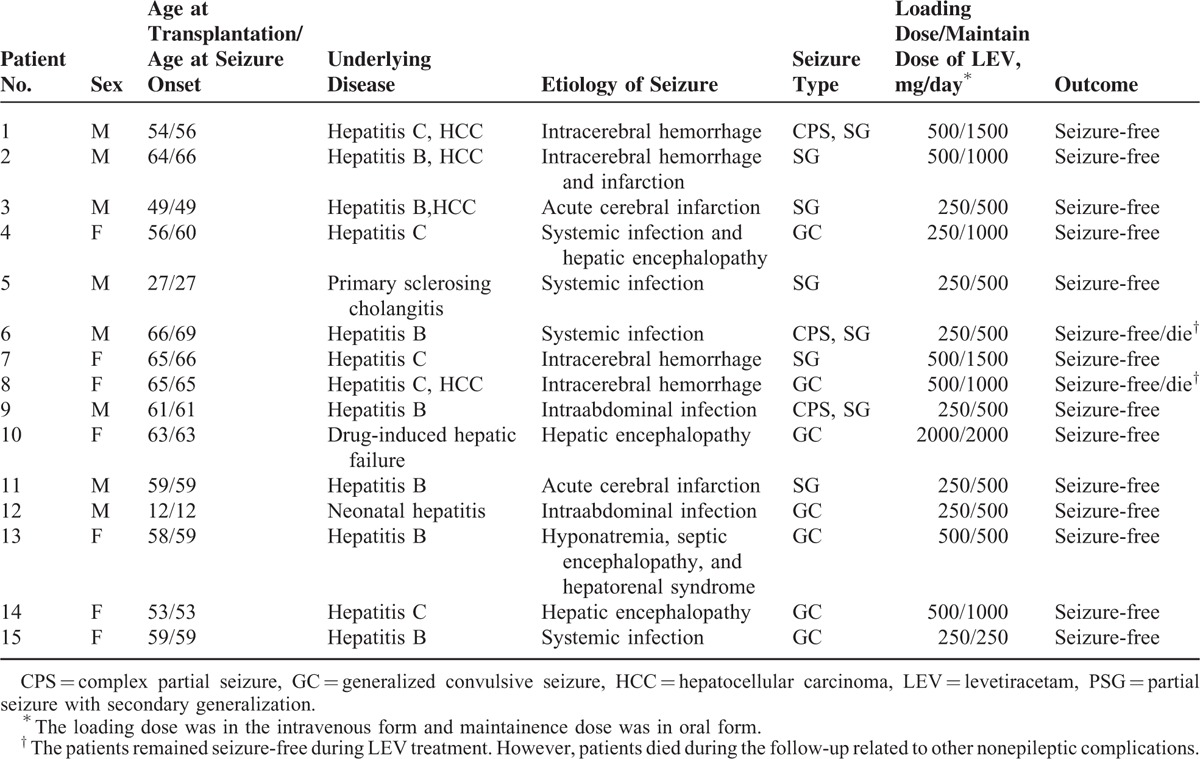
Demographic Data of 15 Patients With Epileptic Seizures After Liver Transplantation

Before using LEV, the white blood cell count was 6.06 ± 4.45  × 10^3^ cells/μL (range from 2.7 to 21.1  × 10^3^ cells/μL); red blood cell count, 3.40 ± 0.73  × 10^6^ cells/μL (range from 2.35 to 4.16  × 10^3^ cells/μL); hemoglobin, 10.0 ± 2.4 g/dL (range from 6.7 to 13.7 g/dL); platelet count, 95.3 ± 34.2 × 10^3^ cells/μL (range from 38 to 135  × 10^3^ cells/μL); serum creatinine, 1.37 ± 1.16 mg/dL (range from 0.54 to 4.46 mg/dL); serum alanine transaminase, 80.6 ± 69.6 IU/L (range from 11 to 252 IU/L); serum total bilirubin, 8.9 ± 12.0 mg/dL (range from 0.7 to 32.8 mg/dL); serum direct bilirubin, 5.21 ± 7.23 mg/dL (range from 0.2 to 23.8 mg/dL); serum alkaline phosphatase, 415.8 ± 557.7 IU/L (range from 33 to 1698 IU/L); and serum gamma-glutamyl transferase, 181.8 ± 315.5 IU/L (range from 6 to 1098 IU/L). One month after using LEV, the white blood cell count was 4.3 ± 2.4 × 10^3^ cells/μL (range from 1.4 to 8.6 × 10^3^ cells/μL); red blood cell count 3.46 ± 0.77 × 10^3^ cells/μL (range from 2.40 to 4.99 × 10^3^/μL); hemoglobin 9.7 ± 2.3 g/dL (range from 6.4 to 13.7 g/dL); platelet count 118.1 ± 58.3 × 10^3^ cells/μL (range from 51 to 190 cells/μL); creatinine 1.49 ± 1.42 mg/dL (range from 0.49 to 6.86 mg/dL); alanine transaminase 44.4 ± 34.1 IU/L (range from 10 to 149 IU/L); total bilirubin 6.0 ± 11.0 mg/dL (range from 0.3 to 38.1 mg/dL); direct bilirubin 4.3 ± 8.3 mg/dL (range from 0.1 to 28.6 mg/dL); alkaline phosphatase 424.8 ± 603.1 IU/L (range from 66 to 1689 IU/L); and gamma-glutamyl transferase 111.1 ± 127.3 IU/L (range from 6 to 420 IU/L). A paired-sample *t* test was conducted to evaluate the changes in these hematological and biochemical data, and there were no significant changes in any of the data before and after the use of LEV.

The immunosuppression medication included prednisolone, mycophenolate, tacrolimus, lamivudine, and sirolimus. Immediately after transplantation, the dose of prednisolone was 7.5 ± 4.5 mg/day (range from 5 to 20 mg/day), mycophenolate 791.7 ± 334.3 mg/day (range from 500 to 1500 mg/day), tacrolimus 2.5 ± 1.4 mg/day (range from 1 to 4 mg/day), lamivudine 75.0 ± 35.4 mg/day (range from 50 to 100 mg/day), and sirolimus 1.7 ± 0.6 mg/day (range from 1 to 2 mg/day). At the last outpatient department follow-up visit, the dose of the prednisolone was 2.9 ± 1.4 mg/day (range from 1 to 5 mg/day), mycophenolate 718.8 ± 525.0 mg/day (range from 500 to 2000 mg/day), tacrolimus 2.4 ± 1.6 mg/day (range from 1 to 5.5 mg/day), lamivudine 75.0 ± 35.4 mg (range from 50 to 100 mg), and sirolimus 1.3 ± 0.5 mg/day (range from 1 to 2 mg/day). To evaluate the changes in dosing of the immunosuppressants, a paired-sample *t* test was used. Only the dose of prednisolone showed a statistically significant decrease (*P* = .041), with a mean decrease of 5.2 mg. There were no significant differences in the doses of the other immunosuppressants before and after the use of LEV.

## DISCUSSION

In this study, we found that LEV was effective in controlling epileptic seizures, including acute symptomatic seizures and remote symptomatic epilepsy after liver transplantation. In addition, there were no statistically significant differences in the hematological and biochemical data with the use of LEV. Furthermore, the dosage of immunosuppressants did not change during LEV treatment, and no patients experienced donor liver failure during the follow-up period.

Epileptic seizures including acute symptomatic seizures and remote symptomatic epilepsy have been reported to be a complication after liver transplantation.^1–3,14^ To optimize AED treatment for liver transplant recipients, several factors should be considered when selecting the AED including the possible presence and degree of hepatic or renal dysfunction, pharmacological interactions between the AED and immunosuppressive drugs, and side effects of the AED on the transplanted liver tissue.^6,7^

Currently, only 3 AEDs are used intravenously, including phenytoin, valproic acid, and LEV. Phenytoin and valproic acid are widely used for seizure control after liver transplantation;^14,15^ however, the use of phenytoin has been reported to increase the risk of further damaging the liver.^16^ Valproic acid is primarily metabolized hepatically, and liver damage from this process is a concern,^16–18^ and it has been suggested that its use should be avoided in liver transplantation.^6^ The use of topiramate may cause drug interaction by inhibiting cytochrome P450 2C19 and inducing of cytochrome P450 3A4 in the liver.^19^ Other side effects of topiramate include those caused by carbonic anhydrase inhibitors, such as renal calculus, periorbital tingling, or digit tingling.^20^ Therefore, the use of topiramate may not be a sound choice in patients who underwent liver transplantation. Gabapentin, LEV, pregabalin, vigabatrin, and topiramate involve no or limited hepatic metabolism, and are suggested as the most appropriate AEDs to treat epilepsy in patients who undergo liver transplantation.^8,21^ LEV is available in both intravenous and oral routes, and there is evidence that it is not processed by the liver and therefore causes less hepatotoxicity.^12^ Therefore, LEV may be the preferred AED for use in the control of seizures after liver transplantation.^14,22^

Enzyme-inducing AEDs such as phenytoin, carbamazepine, and phenobarbital exert prominent effects on the hepatic enzyme system and may reduce plasma levels of cyclosporine, tacrolimus, sirolimus, and corticosteroids.^5,13^ To avoid drug–drug interactions, newer-generation AEDs that exert no or a weak effect on the enzyme system may be preferable to treat epilepsy patients who undergo a liver transplantation. The metabolism of LEV does not involve the hepatic cytochrome P450 system and is mostly excreted unchanged in the urine.^8,10^ LEV metabolism takes place in the blood by hydrolysis into 3 pharmacologically inactive metabolites.^23,24^ As most pharmacokinetic interactions occur in the liver, LEV is less likely to be involved in this process,^8^ and this may reduce the possibility of drug interactions in patients using immunosuppressants.^5^ These interactions may result in donor organ failure or may cause side effects due to increases in the drug dosage necessary to compensate for the interactions. In our series, there was a significant decrease in the dosage of prednisolone after follow-up, which may have been due to scheduled tapering. There were no statistically significant differences in the other immunosuppressants after the use of LEV. As none of our patients experienced liver failure or rejection of the donor liver after LEV, our findings support that LEV has little or no interaction with immunosuppressants.^4,5,13^

The loading and maintenance doses of LEV used in our patients ranged widely. One report including patients using LEV for control of poststroke epilepsy reported a maintenance dose of 1000 to 2000 mg/day.^25^ One possible reason for this discrepancy is that the optimal dosing for these patients has yet to be elucidated. Another possibility is that some patients had impaired renal function, and thus the LEV dose was adjusted according to renal function. This emphasizes the need to consider the medical problems of patients receiving treatment for seizures. In this study, we chose an AED with less hepatotoxicity; however, LEV is primarily excreted from the kidney^10^, and the dosage would need to be adjusted in patients with impaired renal function. The serum level of LEV was not recorded in our study. Since the metabolism of LEV is favorable linearly in pharmacokinetics,^26^ the monitoring of LEV levels is not absolutely required. However, whether the serum level of LEV is a contributing factor for the outcome in patients with liver transplantation requires further study.

Thrombocytopenia has been postulated to be a possible side effect of LEV.^27–29^ A retrospective study reported that the incidence of developing thrombocytopenia among inpatients using LEV was 0.1%.^28^ Another study showed that the use of LEV decreased hematological markers, but still within normal limits.^30^ In our patients, there were many confounding factors that may have led to thrombocytopenia, including liver failure, infection, and the use of immunosuppressants. The cut-off lower limit of the platelet count in our hospital is 150 × 10^3^ cells/μL, and thus the mean platelet count of our patients before and after the use of LEV was considered to indicate thrombocytopenia. However, after statistical analysis, no changes in platelet count were noted, and thus the addition of LEV did not alter the platelet count in our patients.

Liver function tests were regularly performed in our patients. The use of AEDs, especially enzyme inducers, has been reported to modestly increase levels of elevate alanine transaminase, alkaline phosphatase, and gamma-glutamyl transferase.^8^ We compared these levels before and after the use of LEV, and no significant changes were noted. This further supports that LEV is safe to use in patients after liver transplantation.

## CONCLUSION

Based on our results, LEV showed a good effect for seizure control after liver transplantation. During the course of treatment, liver function was not compromised and remained in a steady condition. In addition, there was no need to adjust the dose of LEV with the concurrent use of immunosuppressants with regards to concerns over drug interaction. Taken together, we suggest that LEV monotherapy is safe and effective in the control of epileptic seizures after liver translation.
